# Barycentric rational interpolation collocation method for solving two-dimensional linear and nonlinear integro-differential equations

**DOI:** 10.1371/journal.pone.0354246

**Published:** 2026-07-29

**Authors:** Wei Zhang, Liangli Chen

**Affiliations:** 1 School of Mathematics, Southwestern University of Finance and Economics, Chengdu, Sichuan, China; 2 School of Mathematics, Chengdu Normal University, Chengdu, Sichuan, China; University of Anbar, IRAQ

## Abstract

Barycentric rational interpolation is characterized by excellent numerical stability, high approximation accuracy, and strong adaptability to node distribution. In this paper, we propose a high-precision barycentric rational interpolation collocation method to provide approximate solutions for both linear and nonlinear two-dimensional integro-differential equations (2D-IDEs) subject to specified boundary conditions. Firstly, the discrete scheme for 2D-IDEs is derived by employing the two-dimensional barycentric rational interpolation formula and the two-dimensional Gauss-Legendre numerical integration formula. Then, the error estimation of the approximate solution and the convergence of the method are analyzed. Finally, numerical examples are provided to validate the effectiveness and accuracy of the proposed method.

## 1. Introduction

Integro-differential equations find extensive applications across various fields of science and engineering, including population prediction models [1], economics [2], vibration theory [3], population dynamics [4], and physics [5]. Due to the challenges associated with obtaining analytical solutions for most integro-differential equations, there is a growing interest among scholars in the study of their numerical solutions. Consequently, investigating the numerical solutions of integro-differential equations has emerged as a key focus and a prominent topic within computational mathematics. At present, several scholars have proposed effective numerical methods for obtaining numerical solutions to integro-differential equations. Such as collocation method [6,7], wavelet method [8,9], homotopy perturbation method [10], iterative method [11]. The aforementioned methods primarily focus on one-dimensional integro-differential equations, whereas 2D-IDEs present greater complexity. Consequently, their numerical simulation poses significant challenges, and the accuracy of the calculations employed in these methods is seldom addressed. In addition, the theoretical analysis of numerical solutions is also relatively limited.

In this article, we primarily address the following two-dimensional Volterra-Fredholm integro-differential equations


$$\begin{array}{c} \begin{array}{cc} \sum_{\kappa =0}^{\gamma_{\kappa }}\sum_{\iota =0}^{\gamma_{\iota }}\chi_{\kappa \iota }(x,y)\frac{\partial^{\gamma }u(x,y)}{\partial x^{\kappa }\partial y^{\iota }}+ϝ(u(x,y)) & =g(x,y)+\lambda_{1}\int_{a}^{y}\int_{a}^{x}{\bar{k}}_{1}(x,y;s,t;u(s,t))dsdt\\ & +\lambda_{2}\int_{a}^{c}\int_{a}^{c}{\bar{k}}_{2}(x,y;s,t;u(s,t))dsdt,\;(x,y)\in \Omega , \end{array} \end{array}$$ (1)

where $\gamma =\kappa +\iota ,\kappa =0,1,\cdots ,\gamma_{\kappa },\iota =0,1,\cdots ,\gamma_{\iota }$, with $\gamma_{\kappa },\gamma_{\iota }$ being nonnegative integers, the parameters $\lambda_{1},\lambda_{2}$ are constant, *u*(*x*,*y*) is unknown real function in Ω=[a,c]×[a,c], functions $\chi_{\kappa \iota }(x,y)$ and *g*(*x*,*y*) are known continuous functions in Ω, ϝ(u) and ${\bar{k}}_{i}(x,y;s,t;u(s,t)),i=1,2$ are continuous functions defined on W={(x,y,s,t,u):a≤s≤x<c,a≤t≤y<c}, respectively. The functions *u*(*x*,*y*) and *g*(*x*,*y*) are assumed to be sufficiently smooth to guarantee the existence and uniqueness of a solution.

To date, the issues delineated in Problem (1) have been addressed through several numerical approaches, including the differential transformation method [12], the Haar wavelet collocation method [13], and the Adomian decomposition method [14,15]. Polynomial interpolation based on block-pulse functions [16] and triangular functions [17] have been effectively employed to solve 2D-IDEs. The adopted polynomial interpolation is not only straightforward to implement but also supported by a comprehensive theoretical framework. However, owing to the intrinsic rigidity of the polynomial function space, these methods may result in instability in interpolation outcomes and demonstrate relatively limited numerical precision. Therefore, in the approximation of unknown functions, it is necessary to employ a basis function that exhibits high interpolation accuracy and is well-suited for multi-dimensional problems. The barycentric interpolation collocation method, as proposed by Baltensperger and Berrut [18,19], is a numerical technique of the collocation point type that utilizes barycentric interpolation to approximate unknown functions. This method offers several key advantages, including convenient program implementation, excellent numerical stability, and high computational accuracy, rendering it widely adopted in scientific computing applications. For instance, the barycentric rational interpolation collocation method has been successfully applied to solve the Sine–Gordon equation [20, 21], the Allen-Cahn equation [22], the viscoelastic wave equation [23], the time-fractional evolution equations [24], the second-order Volterra integro-differential equation [25], the 2D nonlinear time-dependent partial integro-differential equations [26], the nonlinear fractional pseudo-parabolic equations [27].

Although numerous numerical methods have been developed for solving 2D-IDEs, significant challenges persist—particularly in balancing numerical stability with high-order accuracy when approximating nonlinear terms. This paper introduces a robust numerical scheme based on the barycentric rational interpolation collocation method. Compared with the traditional methods based on polynomial approximation the proposed approach demonstrates superior numerical accuracy and enhanced computational efficiency. A discretization scheme for 2D-IDEs is developed by combining the two-dimensional barycentric rational interpolation formula with the Gauss–Legendre quadrature rule to approximate the integral kernels. This approach yields a unified computational framework applicable to both linear and nonlinear 2D-IDEs. Theoretical analysis is conducted to establish the error analysis of the proposed method. Numerical experiments further demonstrate that the proposed method achieves higher precision compared to the approaches reported in [17] and [28], while requiring substantially fewer degrees of freedom.

The structure of this paper is organized as follows: Section 2 introduces the properties of one-dimensional and two-dimensional barycentric rational interpolant and differential matrix. Section 3 derives the discrete equations of 2D-IDEs by connecting the two-dimensional barycentric rational interpolant with Gauss-Legendre quadrature formula. Section 4 analyzes the error estimation and convergence of the proposed method. Section 5 provides four examples to test numerical accuracy. Section 6 serves as the conclusion of this document.

## 2. Properties of barycentric rational interpolation

This section primarily reviews the computational formulas for both one-dimensional and two-dimensional barycentric rational interpolation. Moreover, the differentiation matrices for one-dimensional and two-dimensional barycentric interpolation are derived, respectively.

### 2.1. Barycentric rational interpolation

Define a function *u*(*x*) over the interval [*a*, *c*], given *n* + 1 strictly ordered interpolation points $x_{i}$ and their corresponding values $u_{i}=u(x_{i})$, for each i=0,1,…,n. Let $p_{i}(x)$ be the polynomials of degree at most *d* which interpolate the *d* + 1 data pairs $\left\{(x_{i},u_{i}),(x_{i+1},u_{i+1}),\ldots ,(x_{i+d},u_{i+d})\right\}$ of *u*(*x*). Then a set of rational interpolation functions can be obtained as


$$\begin{array}{c} r_{n}[u](x)=\sum_{i=0}^{n-d}\lambda_{i}(x)p_{i}(x)/\sum_{i=0}^{n-d}\lambda_{i}(x),\;\lambda_{i}(x)=\frac{(-1{)}^{i}}{\left(x-x_{i})(x-x_{i+1})\cdots (x-x_{i+d}\right)}, \end{array}$$ (2)

where $\lambda_{i}(x)$ is the original rational interpolation weights and *d* is the rational interpolation parameter. Floater and Hormann [29] have demonstrated that if the interpolation function *u*(*x*) possesses sufficient smoothness, then the rational interpolation function (2) exhibits a convergence order of $O(h^{d+1})$, where $h=\max_{0\leq i\leq n-1}(x_{i+1}-x_{i})$. Furthermore, the barycentric rational interpolation weights are defined as follows


$$\begin{array}{c} \omega_{i}=\sum_{\rho \in J_{i}}(-1{)}^{\rho }\prod_{j=\rho ,\;j\neq i}^{\rho +d}\frac{1}{x_{i}-x_{j}}, \end{array}$$

where $J_{i}=\{\rho \in \;I:i-d\leq \rho \leq i,I=\{0,1,\cdots ,n\}\}$ is an index set. Following [29,30], the rational interpolation function (2) can be expressed in the barycentric form. The notation $r_{n}[u](x)$ is replaced by $u_{n}(x)$ for the sake of clarity and distinction.


$$\begin{array}{c} u_{n}(x)=\sum_{i=0}^{n}\varphi_{i}(x)u_{i},\;\varphi_{i}(x)=\frac{\omega_{i}}{x-x_{i}}/\sum_{\rho =0}^{n}\frac{\omega_{\rho }}{x-x_{\rho }}. \end{array}$$ (3)

Here $u_{n}(x)$ is called the barycentric rational interpolation function and $\varphi_{i}(x)$ is called the barycentric rational interpolation basis functions, which satisfy the property $\varphi_{i}(x_{p})=\delta_{pi}$, p=0,1,2,⋯,n, with $\delta_{pi}$ being the Kronecker-delta functions.

According to the definition of one-dimensional barycentric rational interpolation function, we can derive the corresponding definition for two-dimensional barycentric rational interpolation function. Let *u*(*x*,*y*) be a continuous function on domain Ω. Let $a=x_{0}<x_{1}<\cdots <x_{n}=c$ be *n* + 1 distinct nodes along *x* axis, and $a=y_{0}<y_{1}<\cdots <y_{m}=c$ be *m* + 1 distinct nodes along *y* axis. *d*_1_ and *d*_2_ denote the barycentric rational interpolation parameters, where $0\leq d_{1}\leq n,\;0\leq d_{2}\leq m$. For every $i=0,1,\ldots ,n-d_{1},\;j=0,1,\ldots ,m-d_{2}$, define $u_{ij}=u(x_{i},y_{j})$. Then the two-dimensional barycentric rational function can be expressed as follows


$$\begin{array}{c} r_{nm}[u](x,y)=\sum_{i=0}^{n-d_{1}}\lambda_{i}(x)q_{i}(x,y)/\sum_{i=0}^{n-d_{1}}\lambda_{i}(x),\;\lambda_{i}(x)=\frac{(-1{)}^{i}}{\left(x-x_{i})(x-x_{i+1})\cdots (x-x_{i+d_{1}}\right)}, \end{array}$$

where $\lambda_{i}(x)$ is the original rational weights along *x* axis, $q_{i}(x,y)$ is the interpolation polynomials of the function *u*(*x*,*y*) on the node $\left\{(x_{\tilde{i}},y),\;\tilde{i}=i,i+1,\ldots ,i+d_{1}\right\}$ of degree at most *d*_1_. And


$$\begin{array}{c} q_{i}(x,y)=\sum_{j=0}^{m-d_{2}}\lambda_{j}(y)p_{ij}(x,y)/\sum_{j=0}^{m-d_{2}}\lambda_{j}(y),\;\lambda_{j}(y)=\frac{(-1{)}^{j}}{\left(y-y_{j})(y-y_{j+1})\cdots (y-y_{j+d_{2}}\right)}, \end{array}$$

where $\lambda_{j}(y)$ is the original rational weights along *y* axis, $p_{ij}(x,y)$ is the interpolation polynomial of the function $u_{i}(y)=u(x_{i},y)$ on the node $\left\{(x_{\tilde{i}},y_{\tilde{j}}\right)$, $\tilde{j}=j,j+1,\ldots ,j+d_{2}\}$ of degree at most *d*_2_.

According to the definition provided in (3), for a fixed value of *y*, the barycentric interpolation along the *x* direction at the interpolation nodes $x_{0},x_{1},\ldots ,x_{n}$ is given by


$$\begin{array}{c} u(x,y)=\sum_{i=0}^{n}\varphi_{i}(x)u_{i}(y),\;\varphi_{i}(x)=\frac{\omega_{1,i}}{x-x_{i}}/\sum_{\rho_{1}=0}^{n}\frac{\omega_{1,\rho_{1}}}{x-x_{\rho_{1}}}, \end{array}$$ (4)

where $\omega_{1,i}=\sum_{\rho_{1}\in \;J_{i}}(-1{)}^{\rho_{1}}\prod_{l=\rho_{1},l\neq i}^{\rho_{1}+d_{1}}\frac{1}{x_{i}-x_{l}},\;J_{i}=\{\rho_{1}\in \;I:\;i-d_{1}\leq \rho_{1}\leq i,\;I=\{0,1,\ldots ,n\}\}$ and $0\leq d_{1}\leq n$, $\varphi_{i}(x)$ is the barycentric rational interpolation basis functions along *x* axis.

Analogously, for a fixed value of *x*, the barycentric interpolation concerning $u_{i}(y)$ in the *y* direction at the interpolation nodes $y_{0},y_{1},\ldots ,y_{m}$ is as follows


$$\begin{array}{c} u_{i}(y)=\sum_{j=0}^{m}\psi_{j}(y)u_{ij},\;\psi_{j}(y)=\frac{\omega_{2,j}}{y-y_{j}}/\sum_{\rho_{2}=0}^{m}\frac{\omega_{2,\rho_{2}}}{y-y_{\rho_{2}}}, \end{array}$$ (5)

where $\omega_{2,j}=\sum_{\rho_{2}\in \;J_{j}}(-1{)}^{\rho_{2}}\prod_{l=\rho_{2},l\neq j}^{\rho_{2}+d_{2}}\frac{1}{y_{j}-y_{l}},\;J_{j}=\{\rho_{2}\in \;I:\;j-d_{2}\leq \rho_{2}\leq j,\;I=\{0,1,\ldots ,m\}\}$, and $0\leq d_{2}\leq m$, $\psi_{j}(y)$ is the barycentric rational interpolation basis functions along *y* axis.

For the purpose of clarity, we will use $u_{nm}(x,y)$ in place of $r_{nm}[u](x,y)$. Combining (4) with (5), in the node $\left\{(x_{i},y_{j}),\;i=0,1,2,\ldots ,n;j=0,1,2,\ldots ,m\right\}$, the two-dimensional barycentric rational function is expressed as follows


$$\begin{array}{c} u_{nm}(x,y)=\sum_{i=0}^{n}\sum_{j=0}^{m}\varphi_{i}(x)\psi_{j}(y)u_{ij}=\sum_{i=0}^{n}\sum_{j=0}^{m}b_{ij}(x,y)u_{ij}, \end{array}$$ (6)

where $b_{ij}(x,y)=\varphi_{i}(x)\psi_{j}(y)$ are the two dimensional barycentric rational basis functions, which satisfy the following property $b_{ij}(x_{p},y_{q})=\delta_{ip}\delta_{jq}$. For the sake of clarity, we present the following vector representation.


$$\begin{array}{c} B(x,y)=[b_{00}(x,y),\ldots ,b_{0m}(x,y),\ldots ,b_{n0}(x,y),\ldots ,b_{nm}(x,y)]=\Phi (x)⊗\Psi (y), \end{array}$$

where $\Phi (x)=[\varphi_{0}(x),\varphi_{1}(x),\ldots ,\varphi_{n}(x)]$ and $\Psi (y)=[\psi_{0}(y),\psi_{1}(y),\ldots ,\psi_{m}(y)]$, the symbol ⊗ represents the Kronecker product of a matrix. Any arbitrary function *u*(*x*,*y*) can be expressed as


$$\begin{array}{c} u(x,y)\approx u_{nm}(x,y)=B(x,y)U=[\Phi (x)⊗\Psi (y)]U, \end{array}$$

where $U=[u_{00},\ldots ,u_{0m},\ldots ,u_{n0},\ldots ,u_{nm}{]}^{T}$.

### 2.2. Barycentric interpolation differential matrix

From the formula (3), the μ-order derivative function of $u_{n}(x)$ can be derived by differentiating the barycentric rational interpolation basis function. In other words,


$$\begin{array}{c} u_{n}^{\left(\mu \right)}:=u_{n}^{\left(\mu \right)}(x)=\frac{d^{\mu }u(x)}{dx^{\mu }}=\sum_{i=0}^{n}\varphi_{i}^{\left(\mu \right)}(x)u_{i}, \end{array}$$

where μ is a nonnegative integer. Therefore, the μ-order derivative of the function *u*(*x*) at the node $\{x_{p}{\}}_{p=0}^{n}$ is expressed as follows


$$\begin{array}{c} u_{p}^{\left(\mu \right)}:=u^{\left(\mu \right)}(x_{p})=\frac{d^{\mu }u(x_{p})}{dx^{\mu }}=\sum_{i=0}^{n}\varphi_{i}^{\left(\mu \right)}(x_{p})u_{i}=\sum_{i=0}^{n}D_{pi}^{\left(\mu \right)}u_{i}. \end{array}$$

where $D_{pi}^{\left(\mu \right)}=\varphi_{i}^{\left(\mu \right)}(x_{p})$, p=0,1,…,n. Let $u^{\left(\mu \right)}=[u_{0}^{\left(\mu \right)},u_{1}^{\left(\mu \right)},\cdots ,u_{n}^{\left(\mu \right)}{]}^{T}$ denote the column vector of approximate μ-order fractional derivatives at the discrete nodes, and let $u=[u_{0},u_{1},\cdots ,u_{n}{]}^{T}$ represent the corresponding column vector of function values. The matrix $D^{\left(\mu \right)}$ is a μ-order barycentric rational interpolation differential matrix for the unknown function, whose elements are $D_{pi}^{\left(\mu \right)}$. Therefore, the matrix-vector representation of the aforementioned equation is as follows


$$\begin{array}{c} u^{\left(\mu \right)}=D^{\left(\mu \right)}u. \end{array}$$

According to [31], the elements of the first-order barycentric interpolation differential matrix are formulated as


$$\begin{array}{c} D_{pi}^{\left(1\right)}=\left\{ \begin{array}{c} \frac{\omega_{i}/\omega_{p}}{\left(x_{p}-x_{i}\right)}\;p\neq i,\\ -\sum_{p\neq i}\frac{\omega_{i}/\omega_{p}}{x_{p}-x_{i}}\;p=i. \end{array}\right. \end{array}$$ (7)

And the elements of the μ-order barycentric interpolation differential matrix can be obtained as


$$\begin{array}{c} D_{pi}^{\left(\mu \right)}=\left\{ \begin{array}{c} \mu (D_{pp}^{\left(\mu -1\right)}D_{pi}^{\left(1\right)}-\frac{D_{pi}^{\left(\mu -1\right)}}{x_{p}-x_{i}})\;p\neq i,\\ -\sum_{i=0,p\neq i}^{n}D_{pi}^{\left(\mu \right)}\;p=i. \end{array}\right. \end{array}$$ (8)

The following lemma provides an estimate of the approximation error $e^{\left(\mu \right)}(x)=u^{\left(\mu \right)}(x)-u_{n}^{\left(\mu \right)}(x),\mu \in \{0,1,\cdots ,n\}$, where $u^{\left(\mu \right)}(x)$ is the μ-order derivative function of *u*(*x*), and $u_{n}^{\left(\mu \right)}(x)$ denotes the μ-order derivative of the barycentric rational interpolation $u_{n}(x)$.

**Lemma 2.1** *(*[32]) *Given a function* $u(x)\in C^{d+2}[a,c]$ *with* d∈{1,⋯,n}*, and letting* $u_{n}(x)$ *denotes the the* *barycentric rational interpolation defined* *in (3), there exists a nonnegative constant* $c_{r}$ *for sufficiently large n such that*


$$\begin{array}{c} |u^{\left(\mu \right)}(x)-u_{n}^{\left(\mu \right)}(x)|\leq c_{r}h^{d-\mu +1}, \end{array}$$

*where* $h=\max_{0\leq i\leq n-1}(x_{i+1}-x_{i})$, $\{x_{i}{\}}_{i=0}^{n}$ *represent n + 1 distinct interpolation nodes on [a,c].*

The two-dimensional barycentric interpolation differential matrix is derived based on the one-dimensional barycentric interpolation differential matrix formula (8) and the two-dimensional barycentric interpolation formula (6). In a manner analogous to the method employed for determining the μ-order derivative of the function $u_{n}(x)$, we take the derivatives of both components within the two-dimensional barycentric interpolation formula (6). The (μ+υ)-order partial derivative of *u*(*x*,*y*) can be expressed as


$$\begin{array}{c} u^{\left(\mu ,\upsilon \right)}:=u^{\left(\mu ,\upsilon \right)}(x,y)=\frac{\partial^{\mu +\upsilon }u(x,y)}{\partial x^{\mu }\partial y^{\upsilon }}=\sum_{i=0}^{n}\sum_{j=0}^{m}\varphi_{i}^{\left(\mu \right)}(x)\psi_{j}^{\left(\upsilon \right)}(y)u_{ij}. \end{array}$$

The value of the partial derivative at the node $\left(x_{p},y_{q}\right)$ is estimated


$$\begin{array}{c} \begin{array}{cc} u_{pq}^{\left(\mu ,\upsilon \right)}: & =u^{\left(\mu ,\upsilon \right)}(x_{p},y_{q})=\frac{\partial^{\mu +\upsilon }u(x_{p},y_{q})}{\partial x^{\mu }\partial y^{\upsilon }}\\ & =\sum_{i=0}^{n}\sum_{j=0}^{m}\varphi_{i}^{\left(\mu \right)}(x_{p})\psi_{j}^{\left(\upsilon \right)}(y_{q})u_{ij}=\sum_{i=0}^{n}\sum_{j=0}^{m}D_{pi}^{\left(\mu \right)}C_{qj}^{\left(\upsilon \right)}u_{ij}, \end{array} \end{array}$$ (9)

where p=0,1,⋯,n;q=0,1,⋯,m, $D_{pi}^{\left(\mu \right)}=\varphi_{i}^{\left(\mu \right)}(x_{p}),\;C_{qj}^{\left(\upsilon \right)}=\psi_{j}^{\left(\upsilon \right)}(y_{q})$. To enhance clarity, formula (9) can be expressed in vector notation


$$\begin{array}{c} U^{\left(\mu ,\upsilon \right)}=(D^{\left(\mu \right)}⊗C^{\left(\upsilon \right)})U=D^{\left(\mu ,\upsilon \right)}U, \end{array}$$

where $U^{\left(\mu ,\upsilon \right)}=[u_{00}^{\left(\mu ,\upsilon \right)},\ldots ,u_{0m}^{\left(\mu ,\upsilon \right)},\ldots ,u_{n0}^{\left(\mu ,\upsilon \right)},\ldots ,u_{nm}^{\left(\mu ,\upsilon \right)}{]}^{T}$; $D^{\left(\mu ,\upsilon \right)}=(D^{\left(\mu \right)}⊗C^{\left(\upsilon \right)}),$ $D^{\left(\mu \right)}=[D_{pi}^{\left(\mu \right)}{]}_{p,i=0}^{n}$ is the μ-order differentiation matrix formed by barycentric interpolation in the direction *x* on the points $x_{1},x_{2},\cdots ,x_{n}$; $C^{\left(\upsilon \right)}=[C_{qj}^{\left(\upsilon \right)}{]}_{q,j=0}^{m}$ is the υ-order differentiation matrix formed by barycentric interpolation in the direction *y* on the $y_{1},y_{2},\cdots ,y_{m}$; $D_{pi}^{\left(0\right)}=\delta_{pi}$ and $C_{qj}^{\left(0\right)}=\delta_{qj}$, In other words, $D^{\left(0\right)}=I_{n+1}$ and $C^{\left(0\right)}=I_{m+1}$.

With similar analysis of Lemma 2.1, we have an estimation of $e^{\left(\mu ,\nu \right)}(x,y)=u^{\left(\mu ,\nu \right)}(x,y)-u_{nm}^{\left(\mu ,\nu \right)}(x,y),\mu \in \{0,1,\cdots ,n\},\nu \in \{0,1,\cdots ,m\}$, where $u^{\left(\mu ,\nu \right)}(x,y)$ is the (μ+ν)-order derivative function of *u*(*x*,*y*), and $u_{nm}^{\left(\mu ,\nu \right)}(x,y)$ is the (μ+ν)-order derivative function of the barycentric rational interpolation function $u_{nm}(x,y)$.

**Lemma 2.2** *(*[33]) *Given a function* $u(x,y)\in C^{d_{1}+2}[a,c]\times C^{d_{2}+2}[a,c]$ *with* $d_{1}\in \{1,\cdots ,n\}$*,* $d_{2}\in \{1,\cdots ,m\}$*, and letting* $u_{nm}(x,y)$ *denote the the* *barycentric rational interpolation defined* *in (6), there exists a nonnegative constant* $c_{r}$ *for sufficiently large n,m such that*


$$\begin{array}{c} |u^{\left(\mu ,\nu \right)}(x,y)-u_{nm}^{\left(\mu ,\nu \right)}(x,y)|\leq c_{r}(h_{1}^{d_{1}-\mu +1}+h_{2}^{d_{2}-\nu +1}), \end{array}$$

*where* $h_{1}=\max_{0\leq i\leq n-1}|x_{i+1}-x_{i}|$, $h_{2}=\max_{0\leq j\leq m-1}|y_{j+1}-y_{j}|$, $\{x_{i}{\}}_{i=0}^{n}$ *are n + 1 different interpolation nodes on [a,c],* $\{y_{j}{\}}_{j=0}^{m}$ *are m + 1 different interpolation nodes on [a,c].*

## 3. Numerical method

In this section, we discretize the 2D-IDEs using barycentric rational interpolation and present the corresponding computational formulas. We begin by considering the following 2D-IDEs:


$$\begin{array}{c} ℒu(x,y)+{𝒦}_{1}u(x,y)+{𝒦}_{2}u(x,y)=g(x,y),\;(x,y)\in \Omega , \end{array}$$ (10)

where ℒ is the differential operator, whose expression is


$$\begin{array}{cc} ℒu(x,y) & =a_{1}(x,y)\frac{\partial^{2}u(x,y)}{\partial x^{2}}+a_{2}(x,y)\frac{\partial^{2}u(x,y)}{\partial y^{2}}+a_{3}(x,y)\frac{\partial^{2}u(x,y)}{\partial x\partial y}\\ & \;+b_{1}(x,y)\frac{\partial u(x,y)}{\partial x}+b_{2}(x,y)\frac{\partial u(x,y)}{\partial y}+c_{1}(x,y)u^{r_{1}}(x,y), \end{array}$$ (11)

and 𝒦1 and ${𝒦}_{2}$ are the integral operator, whose expression are


$$\begin{array}{c} {𝒦}_{1}u(x,y)=\lambda_{1}\int_{a}^{y}\int_{a}^{x}k_{1}(x,y;s,t)u^{r_{2}}(s,t)dsdt, \end{array}$$ (12)

$$\begin{array}{c} {𝒦}_{2}u(x,y)=\lambda_{2}\int_{a}^{c}\int_{a}^{c}k_{2}(x,y;s,t)u^{r_{3}}(s,t)dsdt, \end{array}$$ (13)

where $a_{\eta }(x,y),\;b_{\iota }(x,y),\;c_{1}(x,y),\;g(x,y),\;\eta =1,2,3,\iota =1,2$ are known continuous functions in Ω, $r_{i},i=1,2,3$ are positive integer. *k*_1_(*x*,*y*;*s*,*t*) and *k*_2_(*x*,*y*;*s*,*t*) are known continuous functions defined on *W*, *u*(*x*,*y*) is an unknown function. The boundary conditions are


$$\begin{array}{c} \left\{ \begin{array}{c} u(a,y)=f_{1}(y),\;\frac{\partial u}{\partial x}(c,y)=f_{1}^{*}(y),\;a\leq y\leq c,\\ u(x,a)=f_{2}(x),\;\frac{\partial u}{\partial y}(x,c)=f_{2}^{*}(x),\;a\leq x\leq c. \end{array}\right. \end{array}$$ (14)

When $r_{i}=1,i=1,2,3$, equation (10) reduces to a linear 2D-IDEs. To address the linear equations (10) through (14), we employ barycentric rational interpolation to approximate the unknown function *u*(*x*,*y*). Subsequently, formula (6) is substituted into equation (10), yielding the following results.


$$\begin{array}{c} ℒu_{nm}(x,y)+{𝒦}_{1}u_{nm}(x,y)+{𝒦}_{2}u_{nm}(x,y)=g(x,y), \end{array}$$ (15)

where


$$\begin{array}{c} \begin{array}{cc} ℒu_{nm}(x,y) & =a_{1}(x,y)\sum_{i=0}^{n}\sum_{j=0}^{m}{\varphi_{i}^{\prime }}^{\prime }(x)\psi_{j}(y)u_{ij}+a_{2}(x,y)\sum_{i=0}^{n}\sum_{j=0}^{m}\varphi_{i}(x){\psi_{j}^{\prime }}^{\prime }(y)u_{ij}\\ & \;+a_{3}(x,y)\sum_{i=0}^{n}\sum_{j=0}^{m}\varphi_{i}^{\prime }(x)\psi_{j}^{\prime }(y)u_{ij}+b_{1}(x,y)\sum_{i=0}^{n}\sum_{j=0}^{m}\varphi_{i}^{\prime }(x)\psi_{j}(y)u_{ij}\\ & \;+b_{2}(x,y)\sum_{i=0}^{n}\sum_{j=0}^{m}\varphi_{i}(x)\psi_{j}^{\prime }(y)u_{ij}+c_{1}(x,y)\sum_{i=0}^{n}\sum_{j=0}^{m}\varphi_{i}(x)\psi_{j}(y)u_{ij},\\ {𝒦}_{1}u_{nm}(x,y) & =\lambda_{1}\int_{a}^{y}\int_{a}^{x}k_{1}(x,y;s,t)\sum_{i=0}^{n}\sum_{j=0}^{m}\varphi_{i}(s)\psi_{j}(t)u_{ij}dsdt,\\ {𝒦}_{2}u_{nm}(x,y) & =\lambda_{2}\int_{a}^{c}\int_{a}^{c}k_{2}(x,y;s,t)\sum_{i=0}^{n}\sum_{j=0}^{m}\varphi_{i}(s)\psi_{j}(t)u_{ij}dsdt. \end{array} \end{array}$$

To evaluate equation (15) at the nodes $\left\{(x_{p},y_{q}),\;p=0,1,2,\ldots ,n;\;q=0,1,2,\ldots ,m\right\}$, we combine the nature of $\varphi_{i}(x_{p})=\delta_{pi},\;\psi_{j}(y_{q})=\delta_{qj}$ with formula (9), yielding the following expression


$$\begin{array}{c} ℒu_{nm}(x_{p},y_{q})+{𝒦}_{1}u_{nm}(x_{p},y_{q})+{𝒦}_{2}u_{nm}(x_{p},y_{q})=g(x_{p},y_{q}), \end{array}$$ (16)

where


$$\begin{array}{c} \begin{array}{cc} ℒu_{nm}(x_{p},y_{q}) & =a_{1}(x_{p},y_{q})\sum_{i=0}^{n}\sum_{j=0}^{m}D_{pi}^{\prime \prime }\delta_{qj}u_{ij}+a_{2}(x_{p},y_{q})\sum_{i=0}^{n}\sum_{j=0}^{m}\delta_{pi}C_{qj}^{\prime \prime }u_{ij}\\ & \;+a_{3}(x_{p},y_{q})\sum_{i=0}^{n}\sum_{j=0}^{m}D_{pi}^{\prime }C_{qj}^{\prime }u_{ij}+b_{1}(x_{p},y_{q})\sum_{i=0}^{n}\sum_{j=0}^{m}D_{pi}^{\prime }\delta_{qj}u_{ij}\\ & \;+b_{2}(x_{p},y_{q})\sum_{i=0}^{n}\sum_{j=0}^{m}\delta_{pi}C_{qj}^{\prime }u_{ij}+c_{1}(x_{p},y_{q})\sum_{i=0}^{n}\sum_{j=0}^{m}\delta_{pi}\delta_{qj}u_{ij},\\ {𝒦}_{1}u_{nm}(x_{p},y_{q}) & =\lambda_{1}\int_{a}^{y_{q}}\int_{a}^{x_{p}}k_{1}(x_{p},y_{q};s,t)\sum_{i=0}^{n}\sum_{j=0}^{m}\varphi_{i}(s)\psi_{j}(t)u_{ij}dsdt,\\ {𝒦}_{2}u_{nm}(x_{p},y_{q}) & =\lambda_{2}\int_{a}^{c}\int_{a}^{c}k_{2}(x_{p},y_{q};s,t)\sum_{i=0}^{n}\sum_{j=0}^{m}\varphi_{i}(s)\psi_{j}(t)u_{ij}dsdt. \end{array} \end{array}$$

When the differential operator (11) has been estimated, the essential aspect of solving equation (16) lies in the numerical evaluation of the integral ${𝒦}_{1}u_{nm}(x,y)$ and ${𝒦}_{2}u_{nm}(x,y)$. Now, let’s introduce the transformation $\sigma (x)=\frac{x+a}{2}+\frac{x-a}{2}\sigma ,$ $\varsigma (y)=\frac{y+a}{2}+\frac{y-a}{2}\varsigma$ with σ,ς∈[−1,1]. The integral operator defined by equation (12) is derived by evaluating formula (6) at the computational nodes $\left(x_{p},y_{q}\right)$, and is explicitly given by


$$\begin{array}{c} \begin{array}{cc} {𝒦}_{1}u_{nm}(x_{p},y_{q}) & =\lambda_{1}\int_{a}^{y_{q}}\int_{a}^{x_{p}}k_{1}(x_{p},y_{q};s,t)\sum_{i=0}^{n}\sum_{j=0}^{m}\varphi_{i}(s)\psi_{j}(t)u_{ij}dsdt\\ & =\lambda_{1}\zeta_{pq}\int_{-1}^{1}\int_{-1}^{1}k_{1}(x_{p},y_{q};\sigma (x_{p}),\varsigma (y_{q}))\sum_{i=0}^{n}\sum_{j=0}^{m}\varphi_{i}(\sigma (x_{p}))\psi_{j}(\varsigma (y_{q}))u_{ij}d\sigma d\varsigma , \end{array} \end{array}$$

where $\zeta_{pq}=\frac{1}{4}(x_{p}-a)(y_{q}-a)$. Next, we employ the Gauss-Legendre quadrature formula to approximate the double integral $K_{1}u_{nm}(x_{p},y_{q})$


$$\begin{array}{c} \begin{array}{cc} {𝒦}_{1}u_{nm}(x_{p},y_{q}) & \approx \lambda_{1}\zeta_{pq}\sum_{k=1}^{N}\sum_{l=1}^{M}\varpi_{k}\varpi_{l}k_{1}(x_{p},y_{q};\sigma_{k}(x_{p}),\varsigma_{l}(y_{q}))\sum_{i=0}^{n}\sum_{j=0}^{m}\varphi_{i}(\sigma_{k}(x_{p}))\psi_{j}(\varsigma_{l}(y_{q}))u_{ij},\\ & ={k_{1}}_{pi}^{qj}u_{ij}, \end{array} \end{array}$$

where $\sigma_{k}(x_{p})=\frac{x_{p}+a}{2}+\frac{x_{p}-a}{2}\sigma_{k}$, $\varsigma_{l}(y_{q})=\frac{y_{q}+a}{2}+\frac{y_{q}-a}{2}\varsigma_{l}$, *N*,*M* are the number of quadrature nodes in the Gauss quadrature formula, $\varpi_{k},\varpi_{l}$ are integral weight, $\sigma_{k},\varsigma_{l}$ are integral point. Similarly, we can derive the discrete representation of the integral ${𝒦}_{2}u_{nm}(x_{p},y_{q})$. We introduce the following matrices


$$\begin{array}{c} \begin{array}{cc} A_{\eta } & =diag(a_{\eta }(x_{0},y_{0}),\ldots ,a_{\eta }(x_{0},y_{m}),\ldots ,a_{\eta }(x_{n},y_{0}),a_{\eta }(x_{n},y_{1}),\ldots ,a_{\eta }(x_{n},y_{m})),\\ B_{\iota } & =diag(b_{\iota }(x_{0},y_{0}),\ldots ,b_{\iota }(x_{0},y_{m}),\ldots ,b_{\iota }(x_{n},y_{0}),b_{\iota }(x_{n},y_{1}),\ldots ,b_{\iota }(x_{n},y_{m})),\\ C_{1} & =diag(c_{1}(x_{0},y_{0}),\ldots ,c_{1}(x_{0},y_{m}),\ldots ,c_{1}(x_{n},y_{0}),c_{1}(x_{n},y_{1}),\ldots ,c_{1}(x_{n},y_{m})), \end{array} \end{array}$$

where η=1,2,3,ι=1,2. The formula (3) can subsequently be expressed in matrix-vector form


$$\begin{array}{c} \begin{array}{cc} ℒU & =\{A_{1}[D^{\left(2\right)}⊗I_{m+1}]+A_{2}[I_{n+1}⊗C^{\left(2\right)}]+A_{3}[D^{\left(1\right)}⊗C^{\left(1\right)}]+B_{1}[D^{\left(1\right)}⊗I_{m+1}]\\ & \;+B_{2}[I_{n+1}⊗C^{\left(1\right)}]+C_{1}[I_{n+1}⊗I_{m+1}]\}U. \end{array} \end{array}$$

For convenience, let $K_{1}U=[{k_{1}}_{pi}^{qj}]U$, $K_{2}U=[{k_{2}}_{pi}^{qj}]U$, $G=[g_{00},g_{01},\cdots ,g_{0m},\cdots ,g_{n0},g_{n1},\cdots ,g_{nm}{]}^{T}$ with $g_{pq}=g(x_{p},x_{q})$. The equation (16) can be reformulated into the following discrete matrix-vector representation


$$\begin{array}{cc} \left\{A_{1}[D^{\left(2\right)}⊗I_{m+1}\right] & +A_{2}[I_{n+1}⊗C^{\left(2\right)}]+A_{3}[D^{\left(1\right)}⊗C^{\left(1\right)}]+B_{1}[D^{\left(1\right)}⊗I_{m+1}]\;\\ & +B_{2}[I_{n+1}⊗C^{\left(1\right)}]+C_{1}[I_{n+1}⊗I_{m+1}]\}U+K_{1}U+K_{2}U=G. \end{array}$$ (17)

Lastly, we examine discrete boundary conditions. The boundary conditions (14) are discretized utilizing the two-dimensional barycentric rational interpolation formula (6).


$$\begin{array}{cc} u(a,y)=f_{1}(y) & ⟹u_{0q}=f_{1}(y_{q}),\;q=0,1,\ldots ,m,\\ \frac{\partial u}{\partial x}(c,y)=f_{1}^{*}(y) & ⟹\sum_{i=0}^{n}\sum_{j=0}^{m}D_{ni}^{\prime }\delta_{qj}u_{ij}=f_{1}^{*}(y_{q}),\;q=0,1,\ldots ,m,\\ u(x,a)=f_{2}(x) & ⟹u_{p0}=f_{2}(x_{p}),\;p=0,1,\ldots ,n,\\ \frac{\partial u}{\partial y}(x,c)=f_{2}^{*}(x) & ⟹\sum_{i=0}^{n}\sum_{j=0}^{m}\delta_{pi}C_{mj}^{\prime }u_{ij}=f_{2}^{*}(x_{p}),\;p=0,1,\ldots ,n. \end{array}$$ (18)

The aforementioned discrete boundary conditions are incorporated into the relevant positions of the corresponding discrete equation (17). Consequently, we can derive the discrete calculation formula for the barycentric rational interpolation collocation method as applied to 2D-IDEs. The function values at arbitrary non-interpolation points can be evaluated efficiently by


$$\begin{array}{c} U_{nm}(x,y)=\sum_{i=0}^{n}\sum_{j=0}^{m}b_{ij}(x,y)u_{ij}=B(x,y)U, \end{array}$$ (19)

where coefficient matrix $U=[u_{00},\cdots ,u_{0m},\cdots ,u_{n0},\cdots ,u_{nm}{]}^{T}$, and $u_{ij}\approx u(x_{i},y_{j})$.

The nonlinear problems described in equations (10) through (14) can be addressed by a barycentric interpolation Newton-Raphson iterative method. We initially discretize the two-dimensional nonlinear integro-differential equation via barycentric rational interpolation; the resulting system of nonlinear algebraic equations is subsequently solved using the Newton–Raphson iterative method. The discrete nonlinear algebraic equations given in equation (10) can be concisely expressed in matrix form as follows


$$\begin{array}{cc} F(U)= & [A_{1}D^{\left(2,0\right)}+A_{2}D^{\left(0,2\right)}+A_{3}D^{\left(1,1\right)}+B_{1}D^{\left(1,0\right)}+B_{2}D^{\left(0,1\right)}]U\\ & +C_{1}U^{r_{1}}+K_{1}U^{r_{2}}+K_{2}U^{r_{3}}-G=0, \end{array}$$ (20)

The Jacobian matrix corresponding to equation (20) is


$$\begin{array}{c} \begin{array}{cc} F_{d}(U)= & \left[A_{1}D^{\left(2,0\right)}+A_{2}D^{\left(0,2\right)}+A_{3}D^{\left(1,1\right)}+B_{1}D^{\left(1,0\right)}+B_{2}D^{\left(0,1\right)}\right]\\ & +r_{1}C_{1}diag(U^{r_{1}-1})+r_{2}K_{1}diag(U^{r_{2}-1})+r_{3}K_{2}diag(U^{r_{3}-1}), \end{array} \end{array}$$

Based on the Newton–Raphson iterative scheme


$$\begin{array}{c} \begin{array}{c} F_{d}(U_{n-1})U_{n}=F_{d}(U_{n-1})U_{n-1}-F(U_{n-1}),\;n=1,2,3,\cdots , \end{array} \end{array}$$

and the boundary conditions, the iterative procedure yields the numerical solution of equation (20). Regarding the convergence of the Newton–Raphson iteration, it is well known from the standard convergence theory that local quadratic convergence is guaranteed provided that the nonlinear operator F(U) is sufficiently smooth, its Jacobian matrix $F_{d}(U)$ is non-singular and Lipschitz continuous in a neighborhood of the exact solution, and the initial guess $U_{0}$ is sufficiently close to the solution. In our numerical experiments, selecting the zero vector as the initial guess $U_{0}=0$ consistently yields rapid and stable convergence due to the mild nonlinearity of the considered cases. However, it should be noted that a zero initial guess may not be universally appropriate for strongly nonlinear problems, where it could potentially lead to divergence or convergence to unphysical solutions.

## 4. Convergence and error analysis

In this section, we rigorously examine the error bounds and convergence properties between the exact function and the approximate function produced by the barycentric rational interpolation.

**Lemma 4.1** *(*[34]*) For any* $d_{1}\in \{1,\cdots ,n\}$*,* $d_{2}\in \{1,\cdots ,m\}$*,* $u(x,y)\in C^{d_{1}+2,d_{2}+2}(\Omega )$*,* $r_{nm}[u](x,y)$ *is a rational interpolation function of u(x,y), then the interpolation error is*


$$\begin{array}{cc} \left\|u-r_{nm}\right\| & \leq \frac{(n-d_{1})(m-d_{2})h_{1}^{d_{1}+1}}{{\overset{―}{h}}_{1}(d_{1}+1)}\|\frac{\partial^{d_{1}+1}r}{\partial x^{d_{1}+1}}\|+\frac{(n-d_{1})(m-d_{2})h_{2}^{d_{2}+1}}{{\overset{―}{h}}_{2}(d_{2}+1)}\|\frac{\partial^{d_{2}+1}r}{\partial y^{d_{2}+1}}\|\\ & \;+\frac{(n-d_{1})(m-d_{2})h_{1}^{d_{1}+1}h_{2}^{d_{2}+1}}{\left(d_{1}+1)(d_{2}+1\right)}\|\frac{\partial^{d_{1}+d_{2}+2}r}{\partial x^{d_{1}+1}\partial y^{d_{2}+1}}\|\\ & \leq c_{r}h^{d+1}. \end{array}$$ (21)

*where* $h=\min\{h_{1},h_{2}\}$, $h_{1}=\max_{0\leq i\leq n-1}|x_{i+1}-x_{i}|$, $h_{2}=\max_{0\leq i\leq m-1}|y_{i+1}-y_{i}|$, ${\overset{―}{h}}_{1}=\min_{0\leq i\leq n-1}|x_{i+1}-x_{i}|$, ${\overset{―}{h}}_{2}=\min_{0\leq i\leq m-1}|y_{i+1}-y_{i}|$, $d=\min\{d_{1},d_{2}\}$*, and* $c_{r}$ *is a constant.*

Let *u*(*x*,*y*) be the exact solution of (10) and $u_{nm}(x,y)$ is the numerical solution, then we have


$$\begin{array}{c} 𝒯u(x_{p},y_{q})=g(x_{p},y_{q}),\;p=0,1,\cdots ,n;q=0,1,\cdots ,m, \end{array}$$

and


$$\begin{array}{c} \lim_{n,m\to \infty }u_{nm}(x,y)=u(x,y), \end{array}$$

where $𝒯u(x,y)=ℒu(x,y)+K_{1}u(x,y)+K_{2}u(x,y)$. Since *u*(*x*,*y*) is continuous on the compact set Ω, there exists a positive constant $C_{u}$ such that $|u(x,y)|\leq C_{u}$ for all (x,y)∈Ω. Assume that the nonlinear functions $f_{i}(u)=u^{r_{i}}(i=1,2,3)$ satisfy the Lipschitz condition on the interval $\left[-C_{u},C_{u}\right]$, such that


$$\begin{array}{c} |u_{1}^{r_{i}}-u_{2}^{r_{i}}|\leq L_{i}|u_{1}-u_{2}|,\;\forall u_{1}u_{2}\in [-C_{u},C_{u}], \end{array}$$

where $L_{i}$ are the Lipschitz constants. Based on the aforementioned lemma and underlying assumptions, we establish the following theorem.

**Theorem 4.1** *Let* $U^{*}$ *and* U *denote the vectors consisting of the exact values and the numerical approximations at the collocation nodes, respectively. Assuming that the Lipschitz conditions for the nonlinear terms hold. If the integral of the Jacobian matrix* $T=\int_{0}^{1}F_{d}(U+t(U^{*}-U))dt$ *is non-singular and satisfies* $\|T^{-1}{\|}_{\infty }\leq C_{M}$*, then we have*


$$\begin{array}{c} |U_{nm}^{*}(x,y)-U_{nm}(x,y)|\leq C_{E}h^{d-1}. \end{array}$$ (22)

*where* $C_{M}$ *and* $C_{E}$ *is a constant that is independent of h, and* $h=min(h_{1},h_{2})$.

*Proof.* As


$$\begin{array}{c} U_{nm}^{*}(x,y)=\sum_{i=0}^{n}\sum_{j=0}^{m}b_{ij}(x,y)u_{ij}^{*},\;U_{nm}(x,y)=\sum_{i=0}^{n}\sum_{j=0}^{m}b_{ij}(x,y)u_{ij}, \end{array}$$

where $u_{ij}^{*}=u(x_{i},y_{j})$, we have


$$\begin{array}{c} U_{nm}^{*}(x,y)-U_{nm}(x,y)=B(x,y)(U^{*}-U), \end{array}$$ (23)

where $U=[u_{00},u_{01},\ldots ,u_{nm}{]}^{T}$ is the vector of numerical solutions obtained at the nodes $\left(x_{i},y_{j}\right)$, $U^{*}=[u_{00}^{*},u_{01}^{*},\ldots ,u_{nm}^{*}{]}^{T}$ is the vector of exact values at the corresponding nodes, and $B(x,y)=[b_{00}(x,y),\ldots ,b_{0m}(x,y),\ldots ,b_{n0}(x,y),\ldots ,b_{nm}(x,y)]$ is a row vector of size 1×(n+1)(m+1) containing the rational interpolation basis functions. As shown in the derivation of the discrete formulation (20) outlined in Section 3, the numerical solution satisfies the nonlinear algebraic system


$$\begin{array}{c} F(U)=0. \end{array}$$

Substituting the exact solution vector $U^{*}$ into the discrete scheme yields the local truncation error vector τ, such that


$$\begin{array}{c} F(U^{*})=\tau . \end{array}$$

To rigorously map the error of the nonlinear discrete system, we utilize the exact integral form of the Mean Value Theorem for vector-valued functions


$$\begin{array}{c} F(U^{*})-F(U)=\left(\int_{0}^{1}F_{d}(U+t(U^{*}-U))dt\right)(U^{*}-U), \end{array}$$

where $F_{d}$ denotes the Jacobian matrix of the nonlinear system. By defining the intermediate matrix $T=\int_{0}^{1}F_{d}(U+t(U^{*}-U))dt$, we obtain


$$\begin{array}{c} T(U^{*}-U)=\tau ⟹U^{*}-U=T^{-1}\tau . \end{array}$$ (24)

By combining equations (23) and (24), The resulting expression is derived as follows


$$\begin{array}{c} U_{nm}^{*}(x,y)-U_{nm}(x,y)=B(x,y)(U^{*}-U)=B(x,y)T^{-1}\tau . \end{array}$$

Defining a new row vector $M(x,y)=B(x,y)T^{-1}$, the error is rewritten as


$$\begin{array}{c} U_{nm}^{*}(x,y)-U_{nm}(x,y)=M(x,y)\tau . \end{array}$$ (25)

To estimate the upper bound of the truncation error vector τ, we evaluate the continuous truncation error 𝒯e(x,y). Let $e(x,y)=u(x,y)-u_{nm}(x,y)$. Due to the nonlinearity of the integral operators, we utilize the prescribed Lipschitz conditions. For the nonlinear integral operator ${𝒦}_{1}$, the error bound is explicitly given by


$$\begin{array}{c} \begin{array}{cc} \left|{𝒦}_{1}(u)-{𝒦}_{1}(u_{nm})\right| & =\left|\lambda_{1}\int_{a}^{y}\int_{a}^{x}k_{1}(x,y;s,t)[u^{r_{2}}(s,t)-u_{nm}^{r_{2}}(s,t)]dsdt\right|\\ & \leq |\lambda_{1}|\int_{a}^{y}\int_{a}^{x}|k_{1}(x,y;s,t)|\cdot L_{2}|e(s,t)|dsdt\\ & \leq C_{k1}h^{d+1}, \end{array} \end{array}$$

where $C_{k1}$ is a constant depending on $\lambda_{1}$, *L*_2_ is the Lipschitz constant. Similarly, $|{𝒦}_{2}(u)-{𝒦}_{2}(u_{nm})|\leq C_{k2}h^{d+1}$. Applying Lemma 2.2 to the second-order derivatives (i.e., μ=2 or ν=2) in the differential operator ℒ yields a truncation error bounded by $O(h^{d-1})$. Consequently, $|ℒu-ℒu_{nm}|\leq C_{L}h^{d-1}$. In contrast, the nonlinear integral operators introduce an error bounded by $O(h^{d+1})$ due to the Lipschitz conditions. Since 0 < *h* < 1, the lower-order term $O(h^{d-1})$ strictly dominates the overall truncation error. Therefore, the higher-order error $O(h^{d+1})$ from the integral operators does not govern the global rate and is absorbed, leading to the final global error bound of $O(h^{d-1})$. Thus, the components of the truncation error vector τ are bounded by


$$\begin{array}{c} \|\tau {\|}_{\infty }\leq C_{L}h^{d-1}+C_{k1}h^{d+1}+C_{k2}h^{d+1}\leq C_{\tau }h^{d-1}. \end{array}$$

Taking the infinity norm on both sides of the error equation (25) yields


$$\begin{array}{c} \|U_{nm}^{*}-U_{nm}{\|}_{\infty }=\|M(x,y)\tau {\|}_{\infty }\leq \|M(x,y){\|}_{\infty }\|\tau {\|}_{\infty }. \end{array}$$

Since $\|M(x,y){\|}_{\infty }\leq \|B(x,y){\|}_{\infty }\|T^{-1}{\|}_{\infty }$, and acknowledging that the Lebesgue constant associated with the barycentric rational interpolation is bounded by a constant $C_{B}$ (i.e., $\|B(x,y){\|}_{\infty }\leq C_{B}$), we obtain


$$\begin{array}{c} \|U_{nm}^{*}-U_{nm}{\|}_{\infty }\leq C_{B}C_{M}C_{\tau }h^{d-1}=C_{E}h^{d-1}. \end{array}$$

This completes the proof. □

## 5. Numerical examples

In this section, we present four numerical examples to validate the high accuracy of the barycentric rational interpolation collocation method. The numerical results are obtained using the mathematical software MATLAB 2024b. During the computation, the number of interpolation nodes, the number of quadrature nodes, and the rational interpolation parameters are maintained consistently across various variables, namely *n* = *m*, *N* = *M*, $d=d_{1}=d_{2}$. In order to demonstrate the advantages of the proposed method, we compare the numerical results obtained through our approach with those derived from other numerical techniques. This comparison is illustrated by both the absolute error and the maximum absolute error


$$\begin{array}{c} e^{a}(x,y)=|u(x,y)-u_{nm}(x,y)|,\;e(x,y)=\max_{(x,y)\in \Omega }|e^{a}(x,y)|, \end{array}$$

with *u*(*x*,*y*) being the exact solution and $u_{nm}(x,y)$ being the approximate solution. The Newton-Raphson iteration method employs the following convergence criteria: a tolerance of $10\times 10^{-10}$, a maximum iteration limit of 100, and an initial guess equal to the zero vector.

**Example 1** *(*[35]*) Consider the two-dimensional nonlinear Volterra integro-differential equation*


$$\begin{array}{c} \frac{\partial u^{2}(x,y)}{\partial y^{2}}+u(x,y)-\int_{0}^{y}\int_{0}^{x}(s+\cos t)u^{2}(s,t)dsdt=g(x,y),\;x,y\in [0,1], \end{array}$$ (26)

*where*


$$\begin{array}{c} g(x,y)=\frac{1}{8}x^{4}\sin y\cos y-\frac{1}{8}x^{4}y-\frac{1}{9}x^{3}sin^{3}y, \end{array}$$

*with the supplementary conditions*


$$\begin{array}{c} u(x,0)=0,\;\frac{\partial u}{\partial y}(x,0)=x, \end{array}$$

*and the exact solution of the* equation (26) *is given by* u(x,y)=xsiny.

**Fig 1:**
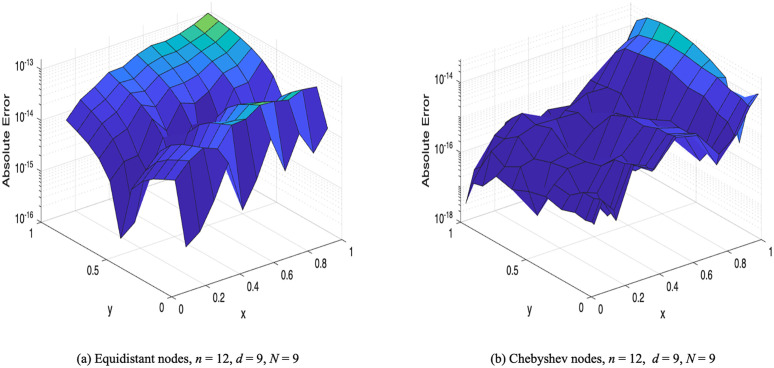
Absolute errors distributions of barycentric rational interpolation collocation method, Example 1.

**Fig 2:**
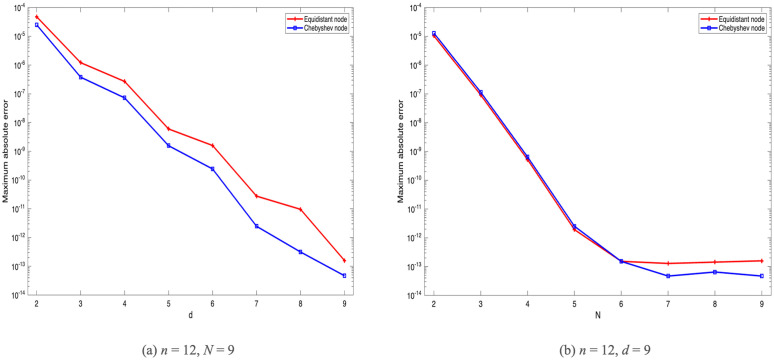
Maximum absolute errors with different parameters, Example 1.

The nonlinear problem (26) is initially reformulated as algebraic equations through the application of barycentric rational interpolation and the Gauss-Legendre quadrature formula. Subsequently, these algebraic equations are solved iteratively using the Newton-Raphson iterative method. Fig 1 illustrates the distributions of absolute error for the barycentric rational interpolation collocation method across various interpolation nodes, with parameters set to *n* = 12, *d* = 9, *N* = 9. This results indicate that the accuracy of the numerical solution is closely related to the choice of interpolation nodes. Fig 2 presents the dependence of the maximum absolute error on the rational interpolation parameter and on the number of quadrature nodes, with *n* fixed at 12. As illustrated in Fig 2, under identical experimental conditions, the maximum absolute error decreases with increasing values of the rational interpolation parameter and with an increasing number of integration nodes. Furthermore, the calculation accuracy of Chebyshev nodes is higher than that of equidistant nodes. Table 1 presents the maximum absolute error associated with *N* = 7, varying the number of interpolation nodes and barycentric rational interpolation parameters. The results indicate that the maximum absolute errors decreases as both the number of interpolation nodes and the barycentric rational interpolation parameters increase.

**Table 1:** Maximum absolute errors of barycentric rational interpolation method, Example 1.

*d*	Equidistant nodes			Chebyshev nodes		
	*n* = 8	*n* = 12	*n* = 16	*n* = 8	*n* = 12	*n* = 16
2	1.3979e-04	4.7930e-05	2.1441e-05	9.5190e-05	2.5074e-05	9.5889e-06
4	1.7424e-06	2.6918e-07	6.8233e-08	6.8580e-07	7.2245e-08	7.4145e-09
6	2.2866e-08	1.5930e-09	2.2908e-10	8.5362e-10	2.4333e-10	2.3189e-11
8	3.0671e-10	9.5917e-12	7.7049e-13	9.3542e-12	3.0320e-13	4.5630e-14

**Example 2**. *(*[28]*) Consider the two-dimensional linear Volterra integro-differential equation*


$$\begin{array}{c} \frac{\partial u(x,y)}{\partial x}=g(x,y)+\int_{0}^{y}\int_{0}^{x}s\cos tu(s,t)dsdt,\;x,y\in [0,1], \end{array}$$ (27)

*where*


$$\begin{array}{c} g(x,y)=e^{-x}(xy\sin y+x\cos y+y\sin y+\cos y-y-x-1)-y\sin y-\cos y+1, \end{array}$$

*with the supplementary condition u(0,y)=0, and the exact solution of the* equation (27) *is given by* $u(x,y)=ye^{\left(-x\right)}$.

**Fig 3:**
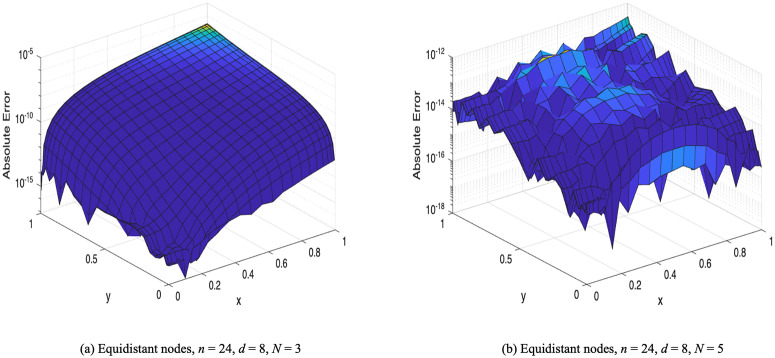
Absolute errors distributions of barycentric rational interpolation collocation method, Example 2.

**Fig 4:**
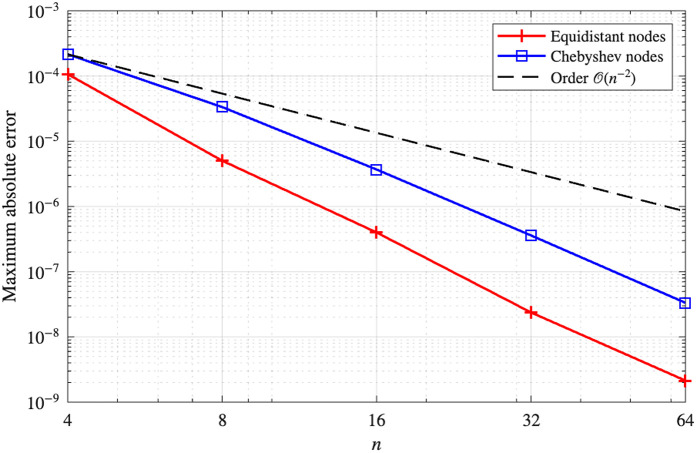
Maximum absolute errors of the barycentric rational interpolation collocation method with *d* = 3, Example 2.

We solve the linear problem (27) by employing the proposed method. Fig 3 illustrates the distributions of absolute error at Equidistant nodes under fixed numbers of interpolation nodes and rational interpolation parameters, computed with varying numbers of quadrature nodes. This indicates that the accuracy of the proposed method is significantly influenced by the selection of quadrature nodal numbers. Fig 4 illustrates the maximum absolute errors as a function of the number of nodes *n*, with fixed parameters *d* = 3 and *N* = 5. It can be observed that the error curves for both equidistant and Chebyshev nodes decrease sharply as *n* increases from 4 to 64. Specifically, the numerical results exhibit a consistent decay rate that aligns perfectly with the reference line of *O*(*n*^-2^). This experimental observation is fully consistent with the theoretical error bound of order $O(h^{d-1})$ established in Theorem 4.1, where *d* = 3 and $h\approx n^{-1}$. Table 2 presents a comparison of the absolute error obtained by the present method and those from the Taylor collocation method [28]. It is clear that the proposed method achieves superior numerical accuracy with a relatively modest number of degrees of freedom. To ensure a fair and unbiased comparison, the absolute errors of the reference methods [17] and [28] are cited directly from their original published papers, whereas the corresponding degrees of freedom (DoF) are calculated by us based on the discretization schemes described in those references.

**Table 2:** Absolute errors of different numerical methods, Example 2.

(*x*,*y*)	Barycentric rational interpolation method			Method in [28]		
	*n* = 10	*n* = 20	*n* = 30	*n* = 10	*n* = 20	*n* = 30
	*DoF* = 121	*DoF* = 441	*DoF* = 961	*DoF* = 1600	*DoF* = 6400	*DoF* = 14400
(0.1,0.1)	2.0767e-11	4.4562e-14	4.6005e-14	7.8981e-07	4.8780e-07	3.4580e-07
(0.2,0.2)	4.0413e-11	1.8788e-13	2.3426e-14	1.4646e-05	7.9986e-06	5.4816e-06
(0.3,0.3)	6.0912e-11	2.0456e-13	3.2224e-14	7.3101e-05	3.8588e-05	2.6178e-05
(0.4,0.4)	8.1221e-11	1.2929e-13	2.2637e-13	2.1930e-04	1.1396e-04	7.6933e-05
(0.5,0.5)	1.0199e-10	2.6973e-13	3.1364e-13	4.9957e-04	2.5728e-04	1.7318e-04
(0.6,0.6)	1.2288e-10	1.9057e-13	3.6393e-13	9.5623e-04	4.8967e-04	3.2902e-04
(0.7,0.7)	1.4452e-10	3.0032e-13	1.6842e-13	1.6220e-03	8.2745e-04	5.5531e-04
(0.8,0.8)	1.6682e-10	2.8877e-13	8.5931e-14	2.5158e-03	1.2801e-03	9.0808e-04
(0.9,0.9)	1.9119e-10	5.1320e-13	1.5182e-13	3.6403e-03	1.8490e-03	1.0290e-03

**Example 3** *(*[36]) *Consider the two-dimensional linear Fredholm integro-differential equation*


$$\begin{array}{c} u(x,y)+\frac{\partial^{2}u(x,y)}{\partial x\partial y}+\int_{-1}^{1}\int_{-1}^{1}(s^{2}-xtsin(y))u(s,t)dsdt=x^{2}-xcos(y)+sin(y)+\frac{4}{5}, \end{array}$$ (28)

*with the supplementary conditions*


$$\begin{array}{c} u(-1,y)=1+cos(y),\;u(x,-1)=x^{2}-xcos(-1), \end{array}$$

*and the exact solution of the equation (28) is given by* $u(x,y)=x^{2}-xcos(y)$.

**Fig 5:**
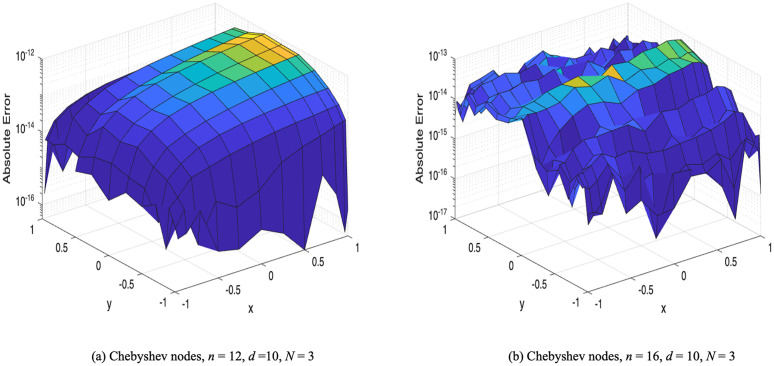
Absolute errors distributions of barycentric rational interpolation collocation method, Example 3.

**Fig 6:**
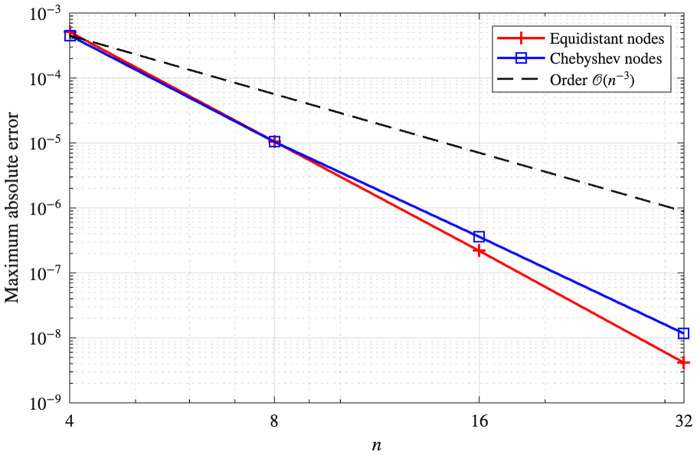
Maximum absolute errors of the barycentric rational interpolation collocation method with *d* = 4, Example 3.

The numerical results of equation (28) utilizing the proposed method are presented in Fig 5,6 and Table 3. Fig 5 demonstrates the absolute error of Chebyshev nodes with varying interpolation nodes counts. The results reveal that when applying fixed rational interpolation parameters and quadrature nodes counts, an increased quantity of interpolation nodes can enhance the accuracy of the numerical solution. Fig 6 displays the log-log plot of the maximum absolute error versus the number of nodes *n* for a fixed *d* = 4, verifying the theoretical convergence rate. It can be clearly observed that the slopes of the error curves for both equidistant and Chebyshev nodes are parallel to the reference line of *O*(*n*^-3^). Notably, the errors obtained using Chebyshev nodes and equidistant nodes are nearly identical at lower degrees, further highlighting the stability and flexibility of the proposed scheme across different node distributions. Table 3 presents the absolute error of the barycentric rational interpolation collocation method, utilizing both Equidistant nodes and Chebyshev nodes, under computational conditions where *n* = 16 and *N* = 5. The numerical results confirm the validity and efficiency of the present method.

**Table 3:** Absolute errors of barycentric rational interpolation collocation method, Example 3.

(*x*,*y*)	Equidistant nodes			Chebyshev nodes		
	*d* = 4	*d* = 6	*d* = 8	*d* = 4	*d* = 6	*d* = 8
(−1.0,-1.0)	5.4579e-13	4.5741e-14	0	5.3291e-15	1.9540e-14	4.4409e-16
(−0.8,-0.8)	8.9846e-09	1.0820e-11	7.8186e-11	3.2527e-09	4.9452e-12	3.8636e-14
(−0.6,-0.6)	2.1630e-08	1.9569e-10	6.3586e-11	3.7582e-08	1.1415e-10	5.4190e-13
(−0.4,-0.4)	3.7736e-08	2.4131e-10	4.5443e-11	6.1018e-08	1.8544e-10	6.3749e-13
(−0.2,-0.2)	2.2765e-08	1.8196e-10	2.1064e-11	1.4812e-07	7.0320e-10	3.3541e-12
(0.0,0.0)	9.2022e-10	7.4176e-11	2.2612e-12	1.6494e-07	7.6588e-10	3.9231e-12
(0.2,0.2)	2.0625e-09	3.6774e-12	2.7819e-11	2.2585e-07	1.0790e-09	5.1484e-12
(0.4,0.4)	1.4324e-08	5.6897e-11	4.9874e-11	1.2816e-07	3.7888e-10	1.2350e-12
(0.6,0.6)	8.0321e-08	1.7791e-10	5.4735e-11	1.0700e-07	3.7526e-10	1.9147e-12
(0.8,0.8)	5.6559e-08	4.2255e-10	5.7680e-11	2.9808e-08	1.9176e-10	1.0753e-12
(1.0,1.0)	2.1871e-07	1.9826e-09	8.2491e-11	3.3352e-08	2.0260e-10	1.1140e-12

**Example 4** *(*[17]*) Consider the two-dimensional nonlinear integro-differential equation*


$$\begin{array}{cc} \frac{\partial^{2}u(x,y)}{\partial x^{2}}+\frac{\partial^{2}u(x,y)}{\partial x\partial y}+u^{3}(x,y)+\int_{0}^{1}\int_{0}^{1}(xy+st^{2})u(s,t)dsdt & \\ +\int_{0}^{y}\int_{0}^{x}(x+y+s+t)[u(s,t){]}^{2}dsdt=g(x,y),\;x,y\in [0,1], \end{array}$$ (29)

*where*


$$\begin{array}{c} \begin{array}{cc} g(x,y)=-\frac{1}{2}xy-\frac{2}{3}+\frac{1}{2}exy+\frac{1}{3}e-\frac{7}{24}x^{4}-\frac{1}{6}x^{3}y+\frac{1}{12}x^{3} & \\ +\frac{7}{24}x^{4}e^{2y}+\frac{1}{3}x^{3}e^{2y}y-\frac{1}{12}x^{3}e^{2y}+e^{y}+x^{3}e^{3y}, \end{array} \end{array}$$

*with the supplementary conditions*


$$\begin{array}{c} u(0,y)=0,\;\frac{\partial u}{\partial x}(0,y)=e^{y}, \end{array}$$

*and the exact solution of the equation (29) is given by* $u(x,y)=xe^{y}$.

**Fig 7:**
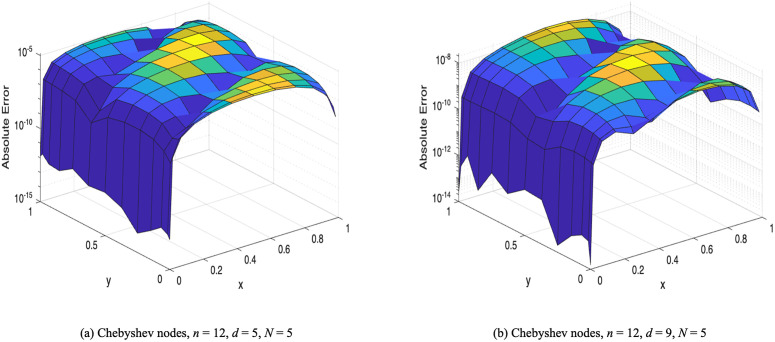
Absolute errors distributions of barycentric rational interpolation collocation method, Example 4.

**Fig 8:**
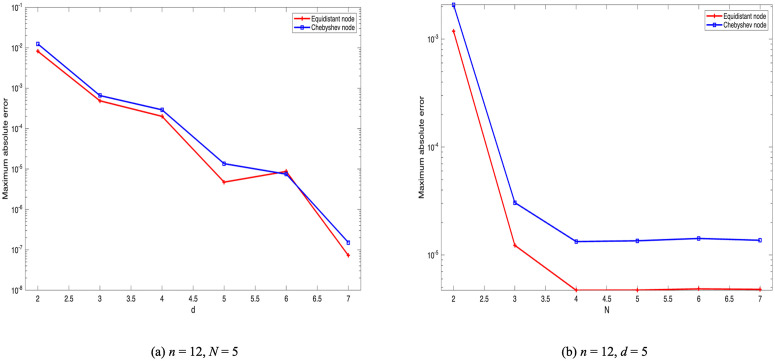
Maximum absolute errors with different parameters, Example 4.

The numerical results obtained using the proposed method to solve equation (29) are presented in Fig 7, 8 and Table 4. Fig 7 depicts the absolute error distributions corresponding to various barycentric rational interpolation parameters, given fixed interpolation nodes and numbers of quadrature nodes. This observation indicates that the numerical accuracy is influenced by the choice of barycentric rational interpolation parameters. Fig 8(a) illustrates the decay of the maximum absolute error as the interpolation degree *d* increases from 2 to 7. Furthermore, Fig 8(b) shows the relationship between the maximum error and the number of nodes *N*. Table 4 shows the numerical comparison results of the proposed method alongside the operational matrix method based on two-dimensional triangular functions as referenced in [17]. It is evident that the proposed method attains higher accuracy while requiring lower computational cost.

**Table 4:** Absolute errors of different numerical methods, Example 4.

(*x*,*y*)	Barycentric rational interpolation method				Method in [17]	
	Equidistant nodes		Chebyshev nodes			
	*n* = 4	*n* = 8	*n* = 4	*n* = 8	*n* = 4	*n* = 8
	*DoF* = 25	*DoF* = 81	*DoF* = 25	*DoF* = 81	*DoF* = 64	*DoF* = 256
(0.1,0.1)	3.1396e-06	3.2281e-09	9.2234e-07	6.3905e-09	1.5322e-02	4.2509e-03
(0.2,0.2)	7.3373e-06	2.7159e-09	1.3782e-05	6.1864e-09	8.4454e-02	1.2145e-03
(0.3,0.3)	1.0649e-05	5.0118e-09	1.3296e-05	4.5813e-09	7.2840e-02	1.2547e-03
(0.4,0.4)	2.3905e-06	1.6640e-08	7.2563e-06	2.1768e-08	5.9522e-02	2.5804e-03
(0.5,0.5)	1.6183e-05	2.7487e-08	2.8828e-05	3.9851e-08	8.7589e-02	3.2154e-03
(0.6,0.6)	1.4829e-05	3.3102e-08	3.4031e-05	5.1616e-08	1.1850e-02	2.2152e-03
(0.7,0.7)	2.2180e-06	3.0896e-08	2.2685e-05	5.2283e-08	2.3698e-02	3.1002e-02
(0.8,0.8)	2.6171e-05	2.0970e-08	3.0762e-06	4.0480e-08	2.5874e-02	7.2425e-02
(0.9,0.9)	4.5712e-05	7.2434e-09	1.9346e-05	1.7309e-08	3.4512e-02	1.9272e-02

## 6. Conclusion

In this paper, we present a collocation method that utilizes barycentric rational interpolation to numerically solve 2D-IDEs subject to specified boundary conditions. The barycentric rational interpolant is employed to discretize the undetermined function. The integro-differential equations are subsequently transformed into systems of algebraic equations through the application of the barycentric interpolation differential matrix and the Gauss-Legendre quadrature formula, along with the implementation of discrete boundary conditions. Furthermore, we conduct a comprehensive analysis of convergence and provide an estimation of the associated errors. The results of numerical examples indicate that the accuracy of calculations using the barycentric rational interpolation collocation method is influenced by several factors, including the type and number of interpolation nodes, the quantity of quadrature nodes, and the selection of parameters for barycentric rational interpolation. The comparison of errors demonstrates that the proposed method exhibits greater accuracy than alternative approaches. Consequently, this method not only provides a strong approximation effect but also ensures high precision. It stands out as a straightforward and effective numerical technique for addressing integral-differential equations.
